# 18β-Glycyrrhetinic acid-loaded silver nanoparticles mitigate neuroinflammation and endoplasmic reticulum stress in the brain tissue of diabetic rats

**DOI:** 10.22038/ijbms.2025.86986.18801

**Published:** 2026

**Authors:** Seçil Nazife Parlak, Seda Yakut, Adem Kara, Özlem Demi̇r, Saime Özbek Şebi̇n

**Affiliations:** 1 Department of Histology and Embryology, Faculty of Medicine, Ağrı İbrahim Çeçen University, Agri, Turkey; 2 Department of Histology and Embryology, Faculty of Veterinary Medicine, Mehmet Akif Ersoy University, Burdur, Turkey; 3 Department of Molecular Biology and Genetics, Faculty of Science, Erzurum Technical University, Erzurum, Turkey; 4 Department of Histology and Embryology, Faculty of Medicine, Erzincan Binali Yildirim University, Erzincan, Turkey; 5 Department of Physiology, Faculty of Medicine, Atatürk University, Erzurum, Turkey

**Keywords:** Apoptosis, Brain, Diabetes mellitus, Endoplasmic reticulum – stress, Glycyrrhetinic acid, Neuroinflammation, Oxidative stress, Silver nanoparticles

## Abstract

**Objective(s):**

Diabetes mellitus (DM) causes oxidative stress, neuroinflammation, and endoplasmic reticulum (ER) dysfunction that contribute to neurodegeneration. This study investigated the effects of 18β-glycyrrhetinic acid-loaded silver nanoparticles (18β-GA-AgNPs) on brain injury in diabetic rats.

**Materials and Methods:**

Fifty-six male Wistar rats were divided into eight groups: Sham, 18β-GA, AgNPs, 18β-GA-AgNPs, DM, DM+18β-GA, DM+AgNPs, and DM+18β-GA-AgNPs. Diabetes was induced by alloxan (120 mg/kg, IP), and treatments were administered orally for 14 days. Biochemical markers (MDA, GSH, SOD), histopathology, and expression of ER stress and apoptotic proteins (ATF6, IRE1, Caspase-3, BCL-2, CREB, TNF-α, and IL-1β) were evaluated.

**Results:**

The DM group exhibited significant increases in MDA, TNF-α, IL-1β, ATF6, and Caspase-3 with reduced GSH, SOD, and BCL-2, indicating oxidative stress, inflammation, apoptosis, and ER stress. In contrast, IRE1 levels remained unchanged in DM rats but showed a slight elevation in the AgNPs group. Treatment with 18β-GA-AgNPs markedly reduced MDA, TNF-α, IL-1β, ATF6, and Caspase-3, while restoring GSH, SOD, BCL-2, and CREB expression. Histopathological analysis confirmed neuronal apoptosis and perivascular and extracellular space enlargement in DM rats, whereas 18β-GA-AgNPs substantially attenuated these changes. Overall, 18β-GA-AgNPs provided synergistic neuroprotection by suppressing oxidative stress, inflammation, and ER stress while enhancing antioxidant and anti-apoptotic defenses.

**Conclusion:**

These findings suggest that 18β-GA-AgNPs may represent a promising therapeutic strategy against diabetes-associated neurodegeneration, although further long-term, ultrastructural, and sex-inclusive studies are warranted.

## Introduction

Diabetes mellitus (DM) is a long-term metabolic disease marked by elevated blood glucose levels due to inadequate insulin production or the body’s reduced sensitivity to insulin (1). Beyond its well-recognized systemic consequences, DM profoundly affects the central nervous system (CNS), leading to disruption of the blood–brain barrier, neuroinflammation, oxidative stress, and endoplasmic reticulum (ER) stress. These processes converge to promote cognitive decline and accelerate the development of neurodegenerative diseases (2, 3). Consequently, there is an urgent need for novel therapeutic strategies that address the interplay between metabolic dysfunction and neuronal injury.

Silver nanoparticles (AgNPs) have attracted considerable attention in biomedical research owing to their distinctive physicochemical features, including small particle size, high surface area-to-volume ratio, and capacity to cross biological barriers such as the blood–brain barrier (4). These attributes render AgNPs promising candidates for drug delivery and therapeutic applications (5-7). AgNPs possess well-documented antimicrobial, anti-oxidant, and anti-inflammatory properties (8-11). Nonetheless, their biological effects are highly context-dependent: while several studies highlight their capacity to attenuate oxidative stress and neuroinflammation, others indicate dose- and size-dependent neurotoxicity mediated by excessive reactive oxygen species (ROS) production, mitochondrial dysfunction, and ER stress (12-15). This dual nature emphasizes the necessity of carefully defining their safe and effective biomedical applications.

18β-Glycyrrhetinic acid (18β-GA), also known as enoxolone, is a bioactive triterpenoid derived from licorice root with established anti-inflammatory, anti-oxidant, antiviral, antimicrobial, and hepatoprotective activities (16, 17). Increasing evidence supports its neuroprotective potential, as it suppresses microglial activation, down-regulates pro-inflammatory cytokine release, and enhances anti-oxidant defense mechanisms. In experimental models of neuroinflammation and ischemic injury, 18β-GA has been associated with reduced neuronal damage and improved functional recovery (18-21). However, its therapeutic role in diabetic brain pathology remains underexplored.

Previous investigations have separately demonstrated the beneficial effects of AgNPs and 18β-GA in diabetic and neurological disease models. AgNPs have been reported to lower blood glucose, modulate oxidative stress, and ameliorate neuroinflammation in diabetic rodents, albeit with concerns regarding long-term safety (22-25). In parallel, 18β-GA has been shown to mitigate neuronal injury by targeting inflammatory and oxidative pathways, though its efficacy in diabetic neuroinflammation has not been systematically evaluated (3, 18, 21, 26). The integration of these two agents, particularly through the formulation of 18β-GA-loaded AgNPs, offers a novel therapeutic strategy. Such an approach may not only improve the bioavailability and targeted delivery of 18β-GA but also counterbalance the potential neurotoxicity associated with free AgNPs, thereby enhancing overall therapeutic efficacy.

In light of these considerations, the present study aims to investigate the neuroprotective potential of 18β-GA-loaded AgNPs in mitigating neuroinflammation and ER stress in the brain tissue of rats with experimentally induced diabetes. By addressing both the pathological hallmarks of diabetic neurodegeneration and the limitations of current nanotherapeutics, this work seeks to provide a foundation for the development of safer and more effective interventions.

## Materials and Methods

***Synthesis and characterization of 18β-glycyrrhetinic acid-loaded silver nanoparticles***

For the synthesis of AgNPs, 0.0167 g of silver nitrate (AgNO₃) was dissolved in 100 mL of distilled water. Separately, 0.020 g of sodium citrate (Na₃C₆H₅O₇) was dissolved in 20 ml of distilled water. The AgNO₃ solution was heated under stirring until it reached boiling. Subsequently, 5 mL of the sodium citrate solution was added dropwise to the boiling AgNO₃ solution, and the mixture was maintained under reflux with continuous stirring for approximately one hour until a visible color change was observed. The reaction mixture was then cooled to room temperature and stored for further use.

**Figure 1:**
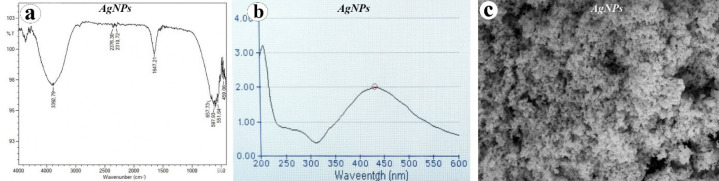
Characterization of silver nanoparticles (AgNPs); (a) The characteristic bands observed in the FTIR spectrum in the range of 3802–669 cm⁻¹ indicate functional groups that play a role in reduction and stability, (b) the prominent surface plasmon resonance (SPR) band around 450 nm in UV-Vis analysis confirms the formation of AgNPs, (c) the SEM image reveals the presence of densely aggregating nanoparticles with spherical morphology.

FTIR: Fourier-transform infrared spectroscopy; SEM: Scanning electron microscopy.

Characterization of the synthesized AgNPs was performed using Ultraviolet–visible (UV–Vis) spectroscopy (Multiskan Sky, Thermo Scientific, Waltham, MA, USA), Fourier transform infrared spectroscopy (FTIR) (Perkin Elmer-Tensor 27, Bruker, Billerica, MA, USA) and scanning electron microscopy (SEM) (FEI Technologies Inc., Hillsboro, OR, USA). FT-IR spectra revealed absorption peaks corresponding to O–H groups at 3287 cm⁻¹, while distinct bands at 1634, 1275, and 1260 cm⁻¹, as well as at 764, 749, 575, and 567 cm⁻¹, were attributed to stretching vibrations of C=O, C=C, and –CH functional groups (Figure 1A). UV–vis spectroscopy showed a strong surface plasmon resonance (SPR) band at around 400 nm, consistent with the characteristic absorbance range reported for AgNPs in the literature (Figure 1B). and SEM analyses. SEM analysis further confirmed the morphology and surface topography of the AgNPs, revealing predominantly spherical particles with sizes ranging from approximately 5 to 100 nm and noticeable agglomeration (Figure 1C).

In the next step, 18β-Glycyrrhetinic Acid (18β-GA) was introduced to the negatively charged citrate-coated AgNPs. The 18β-GA solution was thoroughly mixed with the AgNPs suspension for 3–4 hr, enabling the formation of stable 18β-GA/AgNPs complexes through electrostatic interactions (27).

***Animals and ethical approval***

The Local Animal Ethics Committee of Bingöl University accepted this study under decision number 03/02 during the meeting numbered 2023/03 on May 5, 2023. The animals used in this study included adult male Wistar rats (n=56) obtained from Bingöl University’s Experimental Research Centre. They were aged 6 months and weighed from 220 to 260 g **(**28). The experimental set-up was maintained in a controlled environment with a temperature of 22 **°**C and a 12-hour light and dark cycle. The rats were allowed free access to food and water. The study protocols described complied with the National Institutes of Health Guide for the Care and Use of Laboratory Animals and were approved by the University Ethics Committee **(**29).

***Experimental groups and induction of diabetes***

The experimental subjects were grouped into the following: The Sham group received a single intraperitoneal injection of saline (to mimic alloxan administration) and oral saline daily for 14 days (to mimic treatment protocols) (30). Experimental groups received treatments as follows: The 18 β-GA group was given 100 mg/kg of 18β-GA by oral gavage for 14 days. An oral dose of 100 mg/kg/day of 18β-GA has been widely used in rats for its anti-inflammatory and anti-oxidant effects and has been reported to be safe (31). The AgNPs group was treated with AgNPs 1 mg/kg by oral gavage for 14 days. In animal studies, an oral dose of 1 mg/kg/day of AgNPs has been considered biocompatible and exhibits a low risk of toxicity, while the literature documents dose ranges from 0.25 to 10 mg/kg (32). The 18β-GA-AgNPs group received oral administration of 18β-GA-loaded AgNPs (equivalent to 100 mg/kg 18β-GA and 1 mg/kg AgNPs) for 14 days. In the literature, nanoparticles have been investigated both as drug delivery carriers and as therapeutic agents on their own. AgNPs have been studied for their dual role as carriers as well as for their independent anti-diabetic effects. The rationale for the combination is to enhance the bioavailability of 18β-GA and/or to achieve a synergistic effect (6, 33). Experimental induction of diabetes in rats was performed with a single dose of alloxan monohydrate via intraperitoneal administration following 12 hr of fasting. The alloxan monohydrate was dissolved in normal saline and administered at 120 mg/kg of body weight of rat (34). A dose of 120 mg/kg (IP) is the standard for diabetes induction in studies employing alloxan (35). The presence of diabetes was confirmed by checking blood glucose levels from the collected blood samples. Blood glucose levels were measured using a glucometer and test strips (Accu-Check Active®) from tail vein blood samples. Measurements were taken 1 hr prior to the experiment’s initiation, and subsequently at 24 hr, 48 hr, 72 hr, and 2 weeks after the experiment commenced. Subjects were classified as diabetic and included in the study if their blood glucose levels exceeded 220 mg/dl (36). The DM group received a single intraperitoneal injection of alloxan (120 mg/kg). The DM+18β-GA group received alloxan (120 mg/kg, IP, once) plus oral 18β-GA (100 mg/kg/day) for 14 days. The DM+AgNPs group received alloxan (120 mg/kg, IP, once) plus oral AgNPs (1 mg/kg/day) for 14 days. The DM+18β-GA-AgNPs group received alloxan (120 mg/kg, IP, once) followed by oral administration of 18β-GA-loaded AgNPs (equivalent to 100 mg/kg 18β-GA and 1 mg/kg AgNPs) for 14 days (Table 1).

***Termination of the study***

At the end of the treatment period, all rats in the experimental groups were euthanized under xylazine (4 mg/kg, IP) and ketamine (40 mg/kg, IP) anaesthesia after 12 hr of overnight fasting, and blood samples were taken intracardially **(**37). After blood collection, brain tissues were dissected. For histopathological examination, half of the brain tissue specimens were preserved in a formaldehyde buffer solution at a concentration of 10% (38). The remaining brain tissue samples were preserved in a -80 °C deep freezer until they were needed for biochemical testing. The blood specimens underwent centrifugation at 5000 rpm for a duration of ten min. Following this process, the resulting serum samples were placed in a deep freezer and maintained at -20 °C until they were needed for the required biochemical tests **(**39).

***Biochemical analysis***

***Blood serum measurements***

Quantification of blood glucose concentrations in the obtained serum specimens was performed utilizing an automated analytical instrument (Beckman Coulter, USA).

*Preparation of tissue homogenate*

The experimental protocol includes homogenizing tissue samples in cooler buffer solutions (0.1 M Tris-EDTA buffer, pH 7.4) at a ratio of 1:10 tissue to buffer. Hereafter, the homogenate was spun in a Remi centrifuge maintained at 4 °C while applying 8,000 g over a period of 30 min **(**40). Afterward, the supernatant was carefully taken out and used for the analyses that followed.

*Biochemical measurements on tissues of the brain*

The quantitative determination of lipid peroxidation (LPO) was carried out using the method for thiobarbituric acid-reaction substance (TBARS)-based spectrophotometric determination of malondialdehyde (MDA) as proposed by Placer *et al*. (1966) (41). Results were quantified as μmol of MDA produced per milligram of protein. The measurement of reduced glutathione concentration was performed according to the procedure described by Paglia *et al*. (1975) (42). This activity depends on the action between GSH and 5,5’-dithio-bis (2-nitro-benzoic acid) (DTNB), leading to the formation of a yellow chromophore. Spectrophotometric analysis at 412 nm will be used for measuring the absorbance of such chromophore. This enzyme activity was assessed by the inhibitory power against nitroblue tetrazolium (NBT, Sigma) reduction and was termed superoxide dismutase (EC 1.15.1.1) (43).

*Histopathological analyses*

Following 48 hr fixation, the brain tissue samples underwent a dehydration process through a graded series of alcohols, starting with 50% and progressing through 70%, 96%, and finally 100% alcohol. This gradual dehydration process is crucial for removing water content. Once dehydration was complete, the tissues were cleared using xylene, which enhances tissue transparency and facilitates the infiltration of paraffin wax (44). The tissues were then embedded in paraffin to form solid blocks, a step essential for providing the necessary support for thin sectioning. Utilizing a Leica RM2125 rotary microtome, brain tissue sections were precisely cut to a thickness of 4 µm from the paraffin-embedded blocks **(**44). These thin sections were carefully transferred onto microscope slides, ensuring they adhered properly without wrinkles or folds. Following this, the prepared slides underwent hematoxylin and eosin (H&E) staining **(**44). The stained specimens were then analyzed and imaged using light microscopy techniques. (Nikon Eclipse i50, Tokyo, Japan). 

***Western blot analysis***

Prior to western blot analysis, the obtained brain tissue samples were maintained at −80 **°**C in an ultra-low temperature freezer **(**45). Brain tissue specimens underwent weighing and cryogenic pulverization in nitrogen gas. Subsequently, the samples were subjected to treatment with radioimmunoprecipitation assay (RIPA) buffer (Ecotech Bio, Turkey), enhanced with protease and phosphatase inhibitors **(**45). Homogenization was carried out for 20 seconds at 30 Hz using a tissue lyser (Qiagen, USA). This enabled the relative expression of proteins i.e., ATF 6, IRE1, Caspase 3, TNF-α, IL1-β, BCL-2, DDIT3 (CHOP) and CREB to be determined. Total protein quantification in the brain tissue samples was carried out by means of a protein assay kit (Pierce BCA, Thermo Sci., USA). Protein, 30 µg, was transferred to a PVDF membrane after separation by 10% SDS-PAGE **(**46). 

The membranes were blocked with 5% bovine serum albumin at room temperature for 90 min, followed by overnight incubation at 4 °C with the appropriate primary antibodies. After washing with TBST, the PVDF membranes were incubated for 90 min at room temperature with horseradish peroxidase-linked secondary antibodies (Santa Cruz, sc-2004/sc-2005). The protein bands were then visualized using enhanced chemiluminescence reagent Western ECL substrate (Thermo, 3405) and analyzed using Image Lab™ Software (Bio-Rad, Hercules, CA, USA) **(**45). 

***Statistical analysis***

Statistical methods were applied to calculate the means and standard deviations for the presentation of the data. The choice of analysis depended on whether a given dataset met the parametric assumptions. Normality of the data distributions was tested using the Shapiro–Wilk test, and homogeneity of variances between groups was assessed with Levene’s test. For datasets meeting the parametric assumptions, one-way analysis of variance (ANOVA) was performed. In cases where these assumptions were not satisfied, the Kruskal–Wallis test was applied as the non-parametric alternative. When the Kruskal–Wallis test indicated significant differences, post hoc multiple comparisons were carried out using the Mann–Whitney U test. Exact *P*-values were calculated for all analyses, and statistical significance was defined as *P*<0.05 (36). All statistical analyses were performed using IBM SPSS Statistics (ver. 20.0) and GraphPad Prism 10.1.

## Results

***Biochemical results***

**Figure 2:**
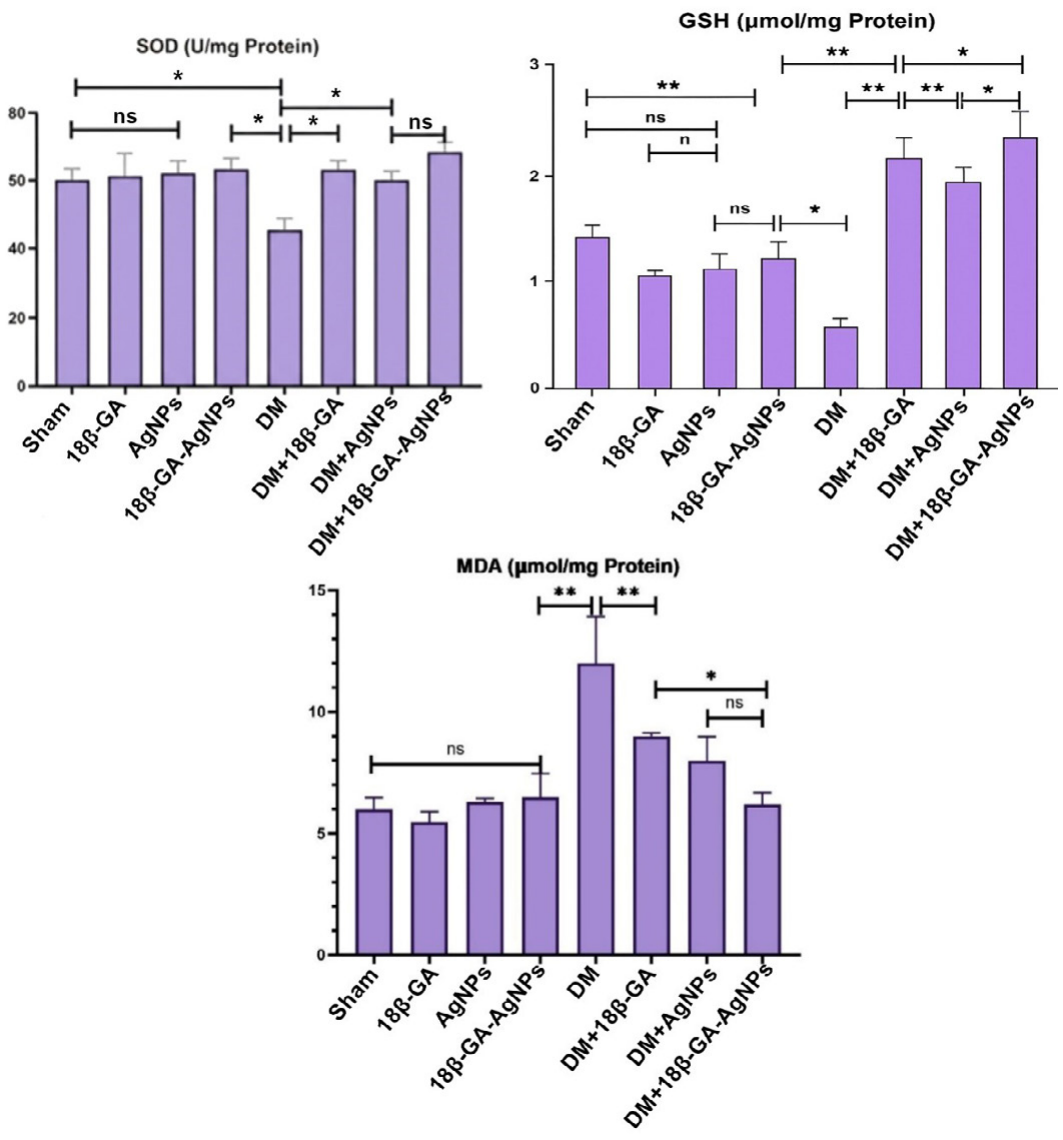
Brain tissue levels of MDA, GSH, and SOD in the experimental groups of male Wistar rats with alloxan-induced diabetes mellitus (mean ± SD, n = 7)

In the biochemical analysis of brain tissues, malondialdehyde (MDA) levels were found to be low in the Sham, 18β-GA, AgNPs, 18β-GA-AgNPs, and DM+18β-GA-AgNPs groups, whereas significantly elevated MDA levels were observed in the DM+18β-GA and DM+AgNPs groups, with the highest levels detected in the DM group (*P*<0.05, Figure 2). Examination of glutathione (GSH) concentrations revealed that the DM group exhibited the lowest GSH levels, while the DM+18β-GA-AgNPs group had the highest concentrations (*P*<0.05). No statistically significant difference was found between the Sham, DM+18β-GA, DM+AgNPs, and DM+18β-GA-AgNPs groups. Furthermore, the 18β-GA and DM groups showed significantly lower GSH levels compared to both the AgNPs and 18β-GA-AgNPs groups (*P*<0.05). Although the Sham group exhibited relatively high GSH concentrations, no significant differences were found compared to the 18β-GA, AgNPs, 18β-GA-AgNPs, DM+AgNPs, and DM groups (ns). Regarding superoxide dismutase (SOD) levels, the DM group showed a statistically significant reduction compared to all other experimental groups (*P*<0.05). No statistically significant differences were found among the Sham, 18β-GA, and AgNPs groups (ns). The DM+18β-GA-AgNPs group exhibited the highest SOD levels, although the difference was not statistically significant when compared to the DM+AgNPs group (ns, Figure 2).

***Histopathological results***

**Figure 3:**
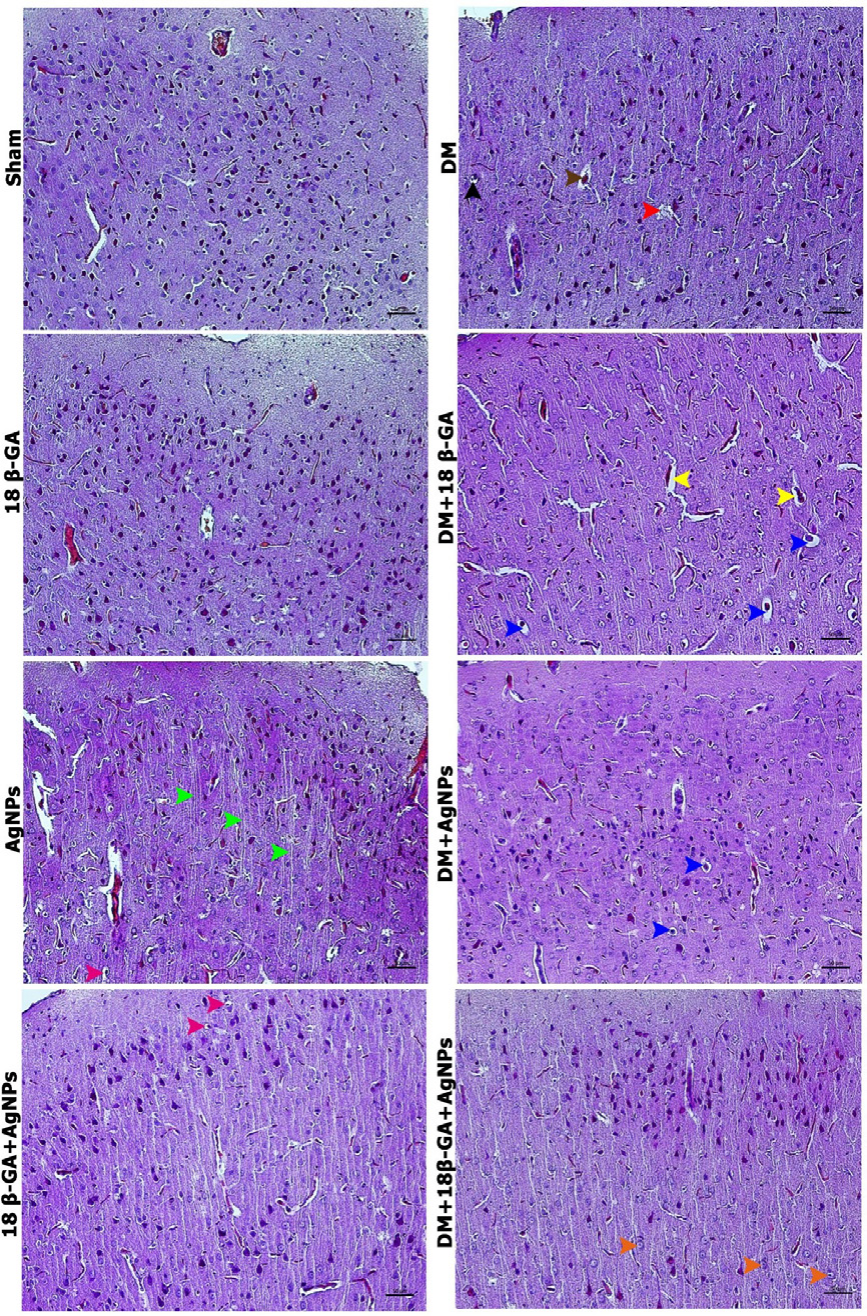
Hematoxylin and eosin staining of brain tissues in all experimental groups of male Wistar rats with alloxan-induced diabetes mellitus, green arrowhead: axon, orange arrowhead: hypertrophic nerve cell, red arrowhead: nuclear fragmentation (apoptotic finding), blue arrowhead: space around nerve cells, yellow arrowhead: space around vessels, brown arrowhead: intense eosinophilic staining of nerve cells (apoptotic finding), pink arrowhead: increased space around supporting cells. Images were acquired using a Nikon Eclipse i50 light microscope at 400× magnification, with scale bars representing 50 μm.

In the Sham group, brain tissues exhibited normal histological features. Similarly, the 18β-GA group displayed histological characteristics consistent with those observed in the Sham group. In the AgNPs group, a notable increase in the number of axons was observed compared to the Sham and 18β-GA groups, along with an expansion of the extracellular space surrounding specific glial cells. In the DM group, neuronal apoptosis and cellular degeneration were prominent in comparison to the Sham and treatment groups. The 18β-GA-AgNPs group showed an increased extracellular space around some supporting glial cells. In both the DM+18β-GA and DM+AgNPs groups, degenerative changes were reduced relative to the DM group. Specifically, the DM+18β-GA group exhibited a pronounced enlargement of the perivascular space, whereas the DM+AgNPs group demonstrated expansion in the space surrounding neurons. Notably, the DM+18β-GA-AgNPs group presented histological features closely resembling those of the Sham group, with a significant reduction in degenerative alterations observed in the DM group. However, slight neuronal hypertrophy was uniquely observed in this group (Figure 3).

***Western blot analysis results***

**Figure 4:**
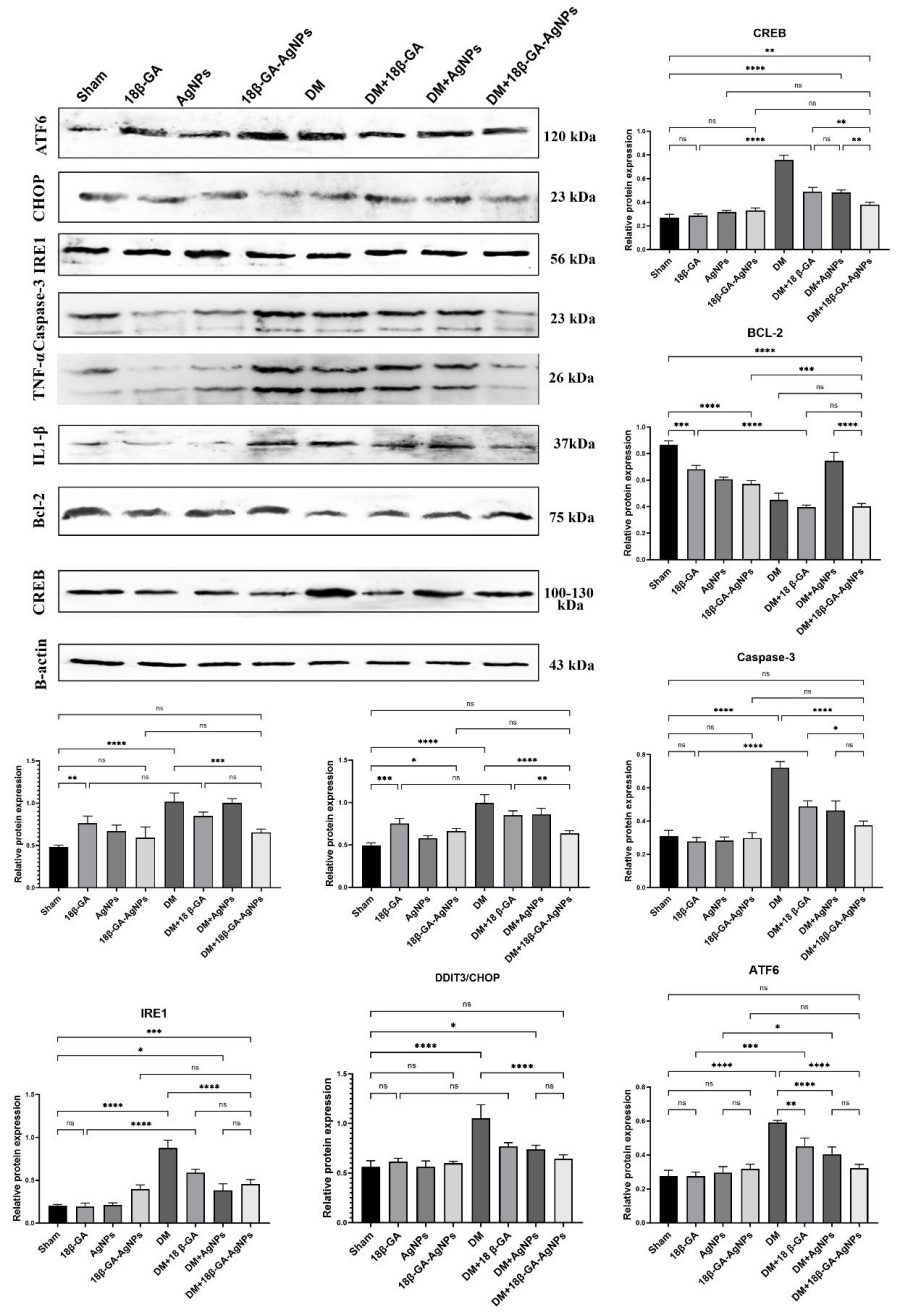
Relative expression levels of endoplasmic reticulum stress, apoptotic, and neuroprotective proteins (ATF6, IRE1, caspase-3, BCL-2, and CREB) in brain tissues of male Wistar rats with alloxan-induced diabetes mellitus across all experimental groups (mean ± SD, n=7)

Examination of brain tissue samples by Western blot analysis revealed that ATF6 expression was significantly increased in the DM and 18β-GA-AgNPs groups compared with the Sham (*P*<0.05). In contrast, the 18β-GA group showed levels closer to the Sham. Caspase-3 levels were markedly elevated in the DM, DM+18β-GA, DM+AgNPs, and 18β-GA-AgNPs groups compared to both the Sham and the 18β-GA group (*P*<0.05). Similarly, TNF-α and IL-1β levels were significantly higher in the DM and combined treatment groups (DM+18β-GA, DM+AgNPs, 18β-GA-AgNPs) compared with the Sham, 18β-GA, and AgNPs groups (*P*<0.05). In contrast, BCL-2 expression was reduced in the DM and combined treatment groups but remained relatively higher in the Sham, 18β-GA, AgNPs, and 18β-GA-AgNPs groups (*P*<0.05). CREB levels were significantly elevated in the DM group, whereas modest increases were observed in the DM+AgNPs and DM+18β-GA-AgNPs groups compared with the Sham (*P*<0.05). IRE1 expression showed a slight, non-significant increase in the AgNPs group, while remaining comparable among the other groups. CHOP expression was elevated in the DM group, with reductions observed in the treatment groups (*P*<0.05). Overall, these findings indicate that DM induces apoptotic and inflammatory signaling, while treatment with 18β-GA, AgNPs, or their combinations modulates these effects to varying degrees (Figure 4). 

## Discussion

This study demonstrated that 18β-glycyrrhetinic acid-loaded AgNPs exert pronounced neuroprotective effects against oxidative stress, neuroinflammation, and endoplasmic reticulum stress in the brain tissue of diabetic rats. By integrating biochemical, histopathological, and molecular findings, our results indicate that this conjugate formulation effectively attenuates diabetes-induced neuronal injury, reinforces endogenous defense mechanisms, and supports neuronal survival.

The elevated MDA levels together with reduced GSH and SOD concentrations observed in the DM group confirm the hallmark oxidative imbalance of diabetic neurodegeneration. (47, 48) Notably, 18β-GA alone did not significantly elevate GSH levels compared to the DM group, suggesting its anti-oxidant potential may be limited without a delivery vehicle. In contrast, the DM+18β-GA-AgNPs group significantly reduced MDA while restoring GSH and SOD levels, thereby re-establishing anti-oxidant defense. These findings align with reports highlighting the anti-oxidant potential of both 18β-GA (49) and biocompatible-dose AgNPs (50). The conjugation appears to maximize therapeutic efficacy while mitigating potential toxicity, as AgNPs alone may exhibit pro-oxidant effects (51) at higher doses or smaller particle sizes.

In the diabetes group, the marked increase of pro-inflammatory cytokines such as TNF-α and IL-1β demonstrated the central role of neuroinflammation in diabetes-related neuronal damage (52). Treatment with 18β-GA-AgNPs significantly reversed these alterations, reducing inflammatory signaling (49, 53). Similarly, the increased expression of Caspase-3 and decreased levels of BCL-2 in the diabetes group confirmed the activation of apoptotic pathways, whereas the conjugate treatment up-regulated BCL-2 and suppressed Caspase-3, thereby supporting neuronal survival (54). However, the moderately elevated levels of TNF-α and caspase-3 observed in the DM+18β-GA-AgNPs group may reflect a transient immune or stress response to nanoparticle exposure and warrant further investigation. Our findings confirmed the ability of 18β-GA to suppress microglial activation and cytokine release (18), while also highlighting the dual nature of AgNPs: anti-inflammatory at low doses but potentially pro-apoptotic at higher concentrations (55).

Up-regulation of ER stress markers ATF6 and IRE1 in the DM group supports the view that ER dysfunction is a key contributor to neurodegeneration in diabetes (56). The 18β-GA-AgNPs treatment effectively reduced ATF6 expression, indicating relief from ER stress. Although IRE1 levels were only mildly altered, the concurrent increase in neuroprotective markers such as BCL-2 and CREB further suggests restoration of ER homeostasis. Notably, the CREB elevation in the DM group may indicate a compensatory stress response rather than genuine neuroprotection, as CREB activation alone does not always translate to functional outcomes (57, 58). Furthermore, the marked elevation of CHOP (C/EBP homologous protein), a key pro-apoptotic factor activated during unresolved ER stress, in the DM group indicates that ER stress-mediated apoptotic pathways are actively engaged in diabetic brain tissue. This increase reflects the failure to restore ER homeostasis under hyperglycemic conditions, ultimately contributing to neuronal loss. Treatment in the DM+18β-GA-AgNPs group significantly reduced CHOP expression, demonstrating a therapeutic effect in suppressing ER stress-induced apoptosis. This reduction suggests that the combined formulation not only alleviates oxidative and inflammatory damage but also mitigates critical apoptotic signaling triggered by prolonged ER stress, thereby reinforcing its neuroprotective potential (59).

The DM group displayed hallmarks of neuronal injury, including apoptosis, perivascular space enlargement, and expansion of extracellular compartments (60, 61). These pathological changes were markedly attenuated in the DM+18β-GA-AgNPs group. Interestingly, neuronal hypertrophy was observed, which may represent either an adaptive compensatory response to injury or an early marker of nanoparticle-induced toxicity (60, 61). Further ultrastructural analyses and long-term toxicity evaluations will be essential to clarify these observations.

One unexpected finding was the low GSH levels in the 18β-GA-only group, highlighting the potential need for a delivery system to unlock the full therapeutic benefit of this compound. Similarly, the histopathological changes in the AgNPs group (e.g., increased axon count and extracellular space) may represent either early neurotoxicity or remodeling.

Moreover, the study only included male rats, thereby precluding analysis of potential sex-based differences in response. Given known sex differences in oxidative stress and neuroinflammation, future studies should incorporate both sexes for broader translational relevance. Finally, the relatively short duration of treatment and lack of behavioral assessments or ultrastructural imaging represent further limitations.

**Table 1:** Experimental groups, treatment protocols, and dosing regimens applied to male Wistar rats with alloxan-induced diabetes mellitus

Group name	n	Applications to be made
Sham	7	Saline (i.p) (single application) + saline (oral gavage) (for 14 days)
18 β-GA	7	18β-GA 100 mg/kg (oral gavage) (for 14 days)
AgNPs	7	Silver Nanoparticle 1 mg/kg (oral gavage) (for 14 days)
18β-GA-AgNPs	7	Oral 18β-GA-loaded silver nanoparticles (equivalent to 100 mg/kg 18β-GA and 1 mg/kg AgNPs) for 14 days
DM	7	Alloxan 120 mg (IP) (single application)
DM+18 β-GA	7	Alloxan 120 mg (IP) (single application) + 18β-GA 100 mg/kg (oral gavage) (for 14 days)
DM+AgNPs	7	Alloxan 120 mg (IP) (single administration) + silver nanoparticle 1 mg/kg (oral gavage) (for 14 days)
DM+18β-GA-AgNPs	7	Alloxan 120 mg (IP) (single administration) + oral 18β-GA-loaded silver nanoparticles (equivalent to 100 mg/kg 18β-GA and 1 mg/kg AgNPs) for 14 days

DM: Diabetes mellitus; AgNPs: Silver nanoparticles; 18β-GA: 18β-glycyrrhetinic acid; IP: Intraperitoneal; n: Number of animals

Statistical analysis was performed using one-way ANOVA followed by Tukey’s post hoc test in GraphPad Prism 10.1. Differences were considered statistically significant at *P*<0.05. *P<0.05; **P<0.01; ns: not significant. MDA: Malondialdehyde; GSH: Glutathione; SOD: Superoxide dismutase; DM: Diabetes mellitus; AgNPs: Silver nanoparticles; 18β-GA: 18β-glycyrrhetinic acid

Protein levels were determined by Western blot and normalized to β-actin. Statistical comparisons were performed using one-way ANOVA and Tukey tests, with *P*<0.05 considered significant. (*); *P*<0.05, (**); *P*<0.01, (***); *P*<0.001, (****); *P*<0.0001, (ns); not significant.

ATF6: Activating transcription factor 6; IRE1: Inositol-requiring enzyme 1; Caspase-3: Cysteine-aspartic protease 3; BCL-2: B-cell lymphoma 2; CREB: cAMP response element-binding protein

## Conclusion

This study shows that 18β-glycyrrhetinic acid-loaded AgNPs protect against diabetes-induced neurodegeneration by reducing oxidative stress, inflammation, and ER stress while enhancing anti-oxidant and anti-apoptotic defenses. The conjugate proved more effective than either compound alone, indicating a synergistic effect. Although limited by short treatment duration, lack of behavioral tests, and absence of ultrastructural data, these findings highlight 18β-GA-AgNPs as a promising therapeutic approach that warrants further long-term and sex-inclusive studies.

## Data Availability

Upon request, researchers will be granted access to the dataset utilized in this study.

## Declaration

We have not used any AI tools or technologies to prepare this manuscript.
